# The Effects of Unfermented and Fermented Cow and Sheep Milk on the Gut Microbiota

**DOI:** 10.3389/fmicb.2019.00458

**Published:** 2019-03-06

**Authors:** Elizabeth A. Rettedal, Eric Altermann, Nicole C. Roy, Julie E. Dalziel

**Affiliations:** ^1^Food Nutrition & Health Team, AgResearch (Grasslands Research Centre), Palmerston North, New Zealand; ^2^Riddet Institute, Massey University, Palmerston North, New Zealand; ^3^The High-Value Nutrition National Science Challenge, Palmerston North, New Zealand

**Keywords:** gut microbiome, fermentation, cow, sheep, cecal microbiota

## Abstract

A variety of fermented foods have been linked to improved human health, but their impacts on the gut microbiome have not been well characterized. Dairy products are one of the most popular fermented foods and are commonly consumed worldwide. One area we currently lack data on is how the process of fermentation changes the gut microbiota upon digestion. What is even less well characterized are the possible differences between cow and other mammals’ milks. Our aim was to compare the impact of unfermented skim milk and fermented skim milk products (milk/yogurt) originating from two species (cow/sheep) on the gut microbiome using a rat model. Male Sprague-Dawley rats were fed a dairy-free diet supplemented with one of four treatment dairy drinks (cow milk, cow yogurt, sheep milk, sheep yogurt) for 2 weeks. The viable starter culture bacteria in the yogurts were depleted in this study to reduce their potential influence on gut bacterial communities. At the end of the study, cecal samples were collected and the bacterial community profiles determined via 16S rRNA high-throughput sequencing. Fermentation status drove the composition of the bacterial communities to a greater extent than their animal origin. While overall community alpha diversity did not change among treatment groups, the abundance of a number of taxa differed. The cow milk supplemented treatment group was distinct, with a higher intragroup variability and a distinctive taxonomic composition. *Collinsella aerofaciens* was of particularly high abundance (9%) for this group. Taxa such as Firmicutes and *Lactobacillus* were found in higher abundance in communities of rats fed with milk, while Proteobacteria, Bacteroidetes, and *Parabacteroides* were higher in yogurt fed rats. *Collinsella* was also found to be of higher abundance in both milk (vs. yogurt) and cows (vs. sheep). This research provides new insight into the effects of unfermented vs. fermented milk (yogurt) and animal origin on gut microbial composition in a healthy host. A number of differences in taxonomic abundance between treatment groups were observed. Most were associated with the effects of fermentation, but others the origin species, or in the case of cow milk, unique to the treatment group. Future studies focusing on understanding microbial metabolism and interactions, should help unravel what drives these differences.

## Introduction

The old adage “You are what you eat,” now appears to accurately reflect the evidence behind the composition and function of the gut microbiome. While other factors such as genetics (Turnbaugh et al., 2009) appear to play a part, environmental factors such as diet have amassed a large amount of evidence as to their influence in building and modifying gut microbial communities (Spor et al., 2011; David et al., 2014; Bokulich et al., 2016). The microbiome has a vital and complex symbiotic relationship with their host, and a fine balance between the two appears necessary to maintain optimal health. Although there are limited studies that concretely prove a cause and effect (i.e., is it microbiota dysbiosis that causes disease or disease that shifts the microbiota), a number of health conditions, including obesity, diabetes, and inflammatory bowel disease (IBD), have been linked to shifts in the gut microbiome (Ley et al., 2005; Frank et al., 2007; Cho and Blaser, 2012). Trying to elucidate what drives and constitutes a balanced gut microbiome and how we can maintain or directionally alter it favorably, has driven research into understanding the effects of food on the gut microbiome.

Fermented foods have a long history of use in humans and have been associated with a number of health benefits (Hata et al., 1996; Kawase et al., 2000; Guyonnet et al., 2007). Consumption of fermented products has been linked to improvements in cholesterol and blood pressure levels, a reduced risk of type 2 diabetes, and reduction in gut irregularity (Chen et al., 2014; Fekete et al., 2015; Cardoso Umbelino Cavallini et al., 2016; Díaz-López et al., 2016; Laatikainen et al., 2016; Nagata et al., 2016). To produce fermented food products, live microorganisms are added to a food, and metabolic activities modify the physical and nutritional characteristics by breaking down complex components and forming by-products.

Although many studies credit the observed health benefits to the live microorganisms in fermented foods, some studies have shown beneficial effects of fermented products containing no viable microorganisms (Ouwehand and Salminen, 1998). These effects have been attributed to the interaction of non-viable bacteria directly with the immune system or the products of microbial fermentation, such as bioactive peptides (Ouwehand and Salminen, 1998). Purified versions of these peptides have been shown to have an effect on bacterial growth (Hartmann and Meisel, 2007; Erdmann et al., 2008) which could potentially modify the composition and function(s) of the gut microbiota.

Yogurt is one of the most commonly consumed fermented foods. Although cow milk is most commonly utilized, a variety of other mammalian milks are available for the commercial production of yogurt. The use of non-bovine milk products has become more popular in Western markets, as people with allergies or sensitivities to cow milk may be able to tolerate non-bovine products (El-Agamy, 2007). The milks across mammalian species also have different nutritional and flavor profiles, which may encourage their consumption.

Currently, we lack an understanding of how consuming fermented foods differs in impact on the gut microbiome from their unfermented forms. Fermentation is known to alter nutritional availability and bioactive compounds, including bioactive peptides, which might be expected to impact the microbiota (Azuma et al., 1984; Liepke et al., 2002). There is also some debate on the extent that fermented foods are capable of affecting gut microbial communities (Veiga et al., 2010, 2014; McNulty et al., 2011). As composition of milk constituents differs among mammals, differences in their effect on the microbiota and pre- and post-fermentation may also occur.

Our aim was to characterize the effects of unfermented and fermented milk (yogurt) originating from cow and sheep on the composition of the gut microbiota. We hypothesized that we would observe differences in bacterial communities due to fermentation, but animal origin would more robustly influence those differences. Rats were fed a solid dairy-free diet supplemented with one of four dairy treatments for 2 weeks. Their cecal bacterial communities were then characterized by looking at diversity, community composition, and correlations with metadata.

## Materials and Methods

The rats used in this study were part of another experiment (parallel) that examined the effect of these dairy treatments on transit time and characterized their peptide profiles and potential bioactivity (Dalziel et al., 2018). Here we separately assessed the cecal contents following the completion of that study. The methods regarding the animal study and dairy drinks are described in Dalziel et al. (2018) but reiterated here for clarity.

### Dairy Drinks Treatments

Cow skim milk powder [SMP 001 (111115)] (38% protein, <0.1% fat, 45% lactose) was donated by NZ Food Innovation Ltd. (Hamilton, New Zealand) while sheep skim milk powder (031215 Cipher number KY03) (52% protein, 1% fat, 37% lactose) was contributed by Blue River Dairy (Invercargill, New Zealand). Powdered milk rather than fresh was used due to the variable availability of large quantities of fresh skimmed sheep milk and for nutrient batch consistency. The pH of the reconstituted cow and sheep milk was measured and found to be 6.5 ± 0.1 for both species. To produce the yogurt, the cow (140 g/L) and sheep (105 g/L) skim milk powders were rehydrated in water using a stick blender for 2 h. Each milk was then slowly heated to 85°C over 2 h and held at that temperature for 30 min, while under constant stirring. The milks were then cooled to 43°C and a starter culture mix (0.26 U/L) of *Streptococcus thermophilus* and *Lactobacillus delbrueckii* ssp. *bulgaricus* (CHR Hansen YF-L811 – YoFlex^®^) was added. This starter culture was chosen for its reliability in producing a consistent product as a drinkable yogurt. Inoculated milks were incubated at 43°C until the pH dropped to 4.5 (4 to 5 h). The yogurts were then frozen at -20°C in shallow trays. To reduce bacterial viability and improve freeze-drying, the yogurts were annealed by thawing to -5°C and refreezing at -20°C before freeze-drying.

The dairy drinks were prepared for usage by reconstituting the milk or yogurt powder to a 3% protein concentration in water and blending for 30 s. Drinks were made up daily and provided as two feeds with half kept at 4°C before use in the animal experiment. A 3% protein concentration was chosen so the yogurt could be delivered as a free-flowing drinkable substance.

To determine the bacterial viability of *S. thermophilus* and *L. delbrueckii* ssp. *bulgaricus* in dairy drinks, milks and yogurts were resuspended in water at 3% protein by blending (30 s). Serial dilutions were performed in phosphate buffered saline (PBS) and 100 μL spread on selective agar plates in triplicate. *S. thermophilus* was grown at 37°C for 24–48 h on Mitis Salivarius agar under 5% CO_2_ conditions. *L. delbrueckii* ssp. *bulgaricus* was grown at 45°C for 72 h on MRS agar (pH 5.2) (Fort Richard Laboratories Ltd, Auckland, New Zealand) under anaerobic conditions. *L. delbrueckii* ssp. *bulgaricus* was not detected in any milk or yogurt samples. *S. thermophilus* was recovered for the cow yogurt (4.5 × 10^6^ CFU/mL) and sheep yogurt (1.5 × 10^4^ CFU/mL), but not recovered from the milks.

### Animal Care and Study Design

The animal study was approved by the AgResearch Grasslands Animal Ethics Committee (Palmerston North, New Zealand) (AE13501) in compliance with the Animal Welfare Act (1999, New Zealand). Forty male Sprague-Dawley rats (initial weight 404 ± 27 g), were obtained from the AgResearch Small Animal Breeding Unit (Hamilton, New Zealand). The rats were individually housed at a constant 21°C with a 12/12 h light/dark cycle. Starting from 10 weeks of age, the rats were fed a dairy-free solid AIN-93M OpenSource Rodent Diet (Research Diets Inc., New Brunswick, NJ, United States) with an egg white protein source. The composition of the rat solid diet is provided in Supplementary Table S1. At 12 weeks of age, one of four dairy drinks [CM, cow milk; CY, cow yogurt; SM, sheep milk; SY, sheep yogurt] were provided *ad libitum* for 14 days (10 rats per group). General health score, weight, and dietary intake were recorded three times a week. Following the completion of the study, the rats were euthanized via CO_2_ overdose inhalation and cervical dislocation. Cecal samples were aseptically collected from rats and immediately stored at -80°C.

### Microbiota Sequencing and Analysis

DNA was extracted from cecal samples that were thawed on ice using the Macherey Nagel Nucleospin Soil kit following the manufacturer’s instructions with the addition of a 5 min bead-beating step (0.6–0.8 mm ceramic beads, FastPrep 120). A NanoDrop^TM^ (Thermo Fisher Scientific) was used to quantify the DNA. DNA samples were then submitted to Omega Bioservices (Norcross, GA, United States) for library preparation and high-throughput sequencing. The libraries were prepared using the Illumina 16S Metagenomic Sequencing kit (Illumina, Inc., San Diego, CA, United States) according to the manufacturer’s protocol. The V3-V4 region of the bacterial 16S rRNA gene was amplified using the 16S Amplicon PCR Forward Primer (5′-TCGTCGGCAGCGTCAGATGTGTATAAGAGACAGCCTACGGGNGGCWGCAG) and 16S Amplicon PCR Reverse Primer (5′-GTCTCGTGGGCTCGGAGATGTGTATAAGAGACAGGACTACHVGGGTATCTAATCC). Amplicon PCR was performed to amplify template out of input DNA samples. Briefly, each 25 μL of polymerase chain reaction (PCR) reaction contained 12.5 ng of sample DNA as input, 12.5 μL 2× KAPA HiFi HotStart ReadyMix (Kapa Biosystems, Wilmington, MA, United States) and 5 μL of 1 μM of each primer. PCR reactions were carried out using the following protocol: an initial denaturation step performed at 95°C for 3 min followed by 25 cycles of denaturation (95°C, 30 s), annealing (55°C, 30 s), extension (72°C, 30 s), and a final elongation of 5 min at 72°C. PCR products were purified using Mag-Bind RxnPure Plus magnetic beads (Omega Bio-tek, Inc., Norcross, GA, United States). A second index PCR amplification, used to incorporate barcodes and sequencing adapters into the final PCR product, was performed in 25 μL reactions, using the same master mix conditions as described above. Cycling conditions were as follows: 95°C for 3 min, followed by eight cycles of 95°C for 30 s, 55°C for 30 s, and 72°C for 30 s. A final 5 min elongation step was performed at 72°C. The libraries were normalized with the Mag-Bind^®^EquiPure Library Normalization Kit (Omega Bio-tek, Inc., Norcross, GA, United States) then pooled. The pooled library ∼600 bases in size was checked using an Agilent 2200 TapeStation and sequenced (2 × 300 bp paired-end read setting) on a MiSeq (Illumina, San Diego, CA, United States).

The sequence data was analyzed using mothur following the methods of Schloss et al. (2009, 2011). Briefly, paired-end reads (3,519,095 pre-quality control paired reads) were assembled and underwent quality control that removed reads containing uncalled bases and homopolymers of greater than eight. The average quality scores across all bases was greater than 30 and the average sequence length was 416 bp. Sequences were then aligned against the SILVA database (release 132) (Quast et al., 2013). Following sequencing alignment and filtering, the sequencing reads underwent a pre-clustering step (4 bp) designed to denoise and reduce the effect of sequencing errors. Chimeric sequences were detected and removed using VSEARCH (Rognes et al., 2016). Following taxonomic classification, non-bacterial sequences were removed from the analysis and sequences were clustered into OTUs at a 97% cutoff. A total of 1,434,251 reads remained following all quality control and filtering steps with an average of 29,880 reads per sample. The number of sequences per sample was subsampled to 17,323 to equalize the number of reads across samples for all downstream analyses. BIOM tables were exported from mothur for use in other analysis programs. Raw output from mothur taxonomic classifications is available in Supplementary Table S2.

Alpha-diversity was calculated using the Shannon index. Taxonomic graphs were generated using classifications from mothur. LEfSe was used to identify taxonomic features significantly different (*p* < 0.05) and most likely to explain the differences in bacterial communities (LDA ≥ 2) (Segata et al., 2011). An OTU most likely species identity was determined by a BLAST nr search (Altschul et al., 1990), utilizing the top hit. Bar chart graphics for taxonomic graphs and LEfSe results were generated using Microsoft Excel^®^. Multiple linear regression analyses were performed using Calypso (Zakrzewski et al., 2017) to determine bacterial OTUs that correlated with previously collected intestinal transit scores (Dalziel et al., 2018). Rats with transit scores of less than 10 (6 rats) were removed from the analysis (2 CY, 2 SM, 2 SY), as it has been previously shown that ∼10% of animals can have significantly delayed stomach emptying resulting in unusable transit data (Dalziel et al., 2016).

### Statistical Analyses

Comparisons between rat dietary intake values and weights were analyzed using a one-way ANOVA. Standard deviations (SD) were also calculated and reported for average intake and body weight increases. PERMANOVA and PERMDISP analyses were perform with Calypso. *P* values less than 0.05 were considered significant.

## Results

### Rat Dietary Intake and Weights

Rats in this study were fed a solid dairy-free diet to remove the potentially confounding effects of other dairy components in the diet. Powdered skimmed milks were used and the dairy drinks were diluted to 3% protein to level the fat and protein intake.

Rats across all treatment groups had an average daily intake of 27 ± 3.5 g (SD) of solid food and body weight increased by 19 ± 3.9% (SD) over 14 days (Supplementary Table S3) (Dalziel et al., 2018). There was no significant difference between daily solid food intake or treatment group weights on Day 0 or 14 (Supplementary Table S3). For the dairy drinks, the CM group (87.48 mL) had the lowest daily mean intake and the CY group the highest (103.73 mL), but the intake difference between all groups was not statistically significant (ANOVA, *p* = 0.07) (Supplementary Table S3).

### Cecal Microbiota Differs Among Dairy Drink Treatments

To determine if there were differences among the four dairy drink treatment groups, the high-throughput sequencing data was analyzed for diversity, community composition, taxonomy, and correlations with metadata. To look at alpha diversity, the Shannon indices were determined. We found no significant differences between the treatment groups (Supplementary Figure S1).

**FIGURE 1:**
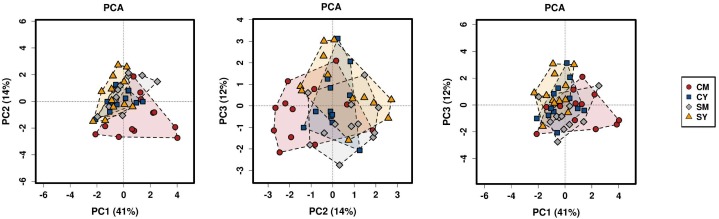
A PCA plot of the differences in bacterial community composition between the four dairy drink treatment groups [CM, cow milk; CY, cow yogurt; SM, sheep milk; SY, sheep yogurt] as calculated using the Bray-Curtis index. The analysis was based on OTU-level taxonomic data.

Next, we determined differences in overall bacterial community composition. A Bray-Curtis principal component analysis (PCA) plot did not show a distinct separation between treatment groups, but rather more of a gradient-like distribution with the cow milk treatment showing the largest spread between individual rats (Figure 1). To determine if there might be a significant difference between groups, PERMANOVA was performed, which suggested a difference between the treatment groups (*p* < 0.001). To look at the possible effects of intragroup variation, PERMDISP was performed which indicated a significant difference (*f* = 4.6861, *p* = 0.0063). The combined results from the PERMANOVA and PERMDISP analyses indicate that while there was a difference between treatment groups, at least some of those differences were due to variance in intragroup variability.

**FIGURE 2:**
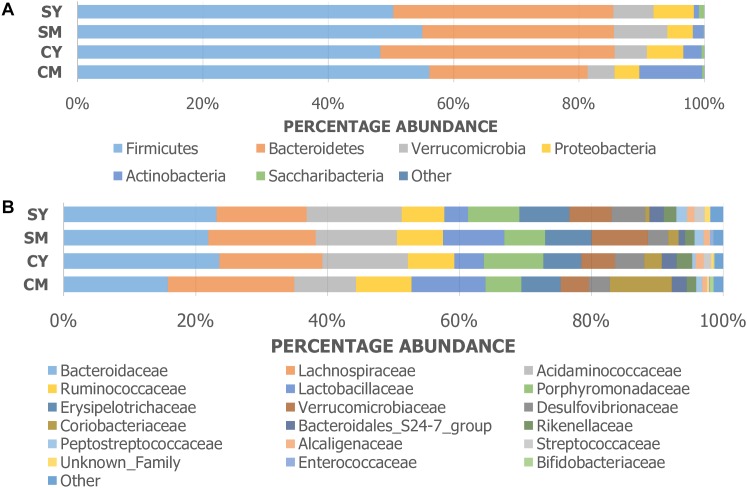
Taxonomic graphs representing the **(A)** phylum and **(B)** family level bacterial communities (group average) found in each dairy drink treatment group [CM, cow milk; CY, cow yogurt; SM, sheep milk; SY, sheep yogurt].

The taxonomic composition of the treatment groups on phylum and family levels are visualized in Figure 2. At phylum level (Figure 2A), the cow milk (CM) group was particularly distinct with increased Actinobacteria and decreased Bacteroidetes. The milks (CM, SM) had similar amounts of Firmicutes while the yogurts (CY, SY) contained comparable ratios of both Firmicutes and Bacteroidetes. At a family-level taxonomic classification (Figure 2B), taxonomic abundance differences could also be seen in specific treatments (e.g., higher *Coriobacteriaceae* in CM) and between unfermented and fermented products (e.g., higher *Lactobacillaceae* in milks). The difference in community intragroup variation noted by PERMDISP analysis, suggested higher taxonomic variability among individual rats in some treatment groups (e.g., CM) (less uniform taxonomic composition). Taxonomic graphs of individual rats highlight these samples (Supplementary Figure S2).

**FIGURE 3:**
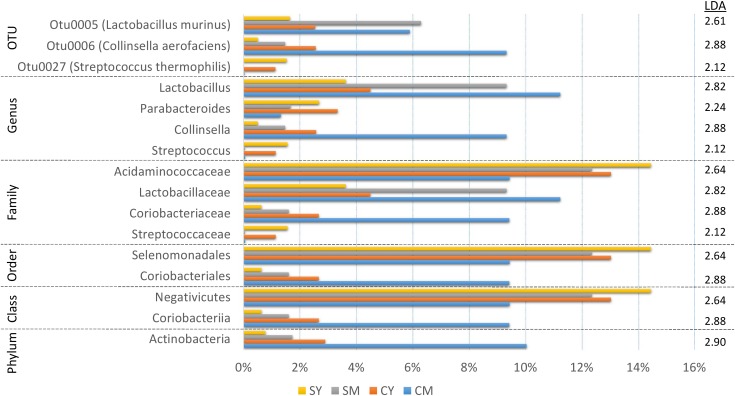
A bar graph of the taxonomic groups indicated by LEfSe analysis to be the most likely to explain the differences between the four dairy drink treatment groups [CM, cow milk; CY, cow yogurt; SM, sheep milk; SY, sheep yogurt] (*p* < 0.05, LDA score ≥ 2). The bars represent the abundance of the taxa found in each treatment group. The LDA scores are listed on the right.

In order to identify robust and meaningful differences in taxonomic abundances between treatment groups, we chose to use LEfSe to identify taxonomic groups with significant differences ranging from phylum to OTU. LEfSe identified 16 taxa with significant changes (Figure 3). While a number of taxa were identified, similar to the taxonomic composition graphs, the CM dairy drink treatment clearly had a more unique taxonomic representation. Its particularly high abundance in *Collinsella aerofaciens* (OTU6) (*Collinsella*, *Coriobacteriaceae*, *Coriobacteriales*, *Coriobacteriia*, Actinobacteria) and lower abundance in *Acidaminococcaceae* (*Selenomonadales*, *Negativicutes*) were absent from the other dairy drink groups.

### Fermentation Status Influences Cecal Microbial Communities

**FIGURE 4:**
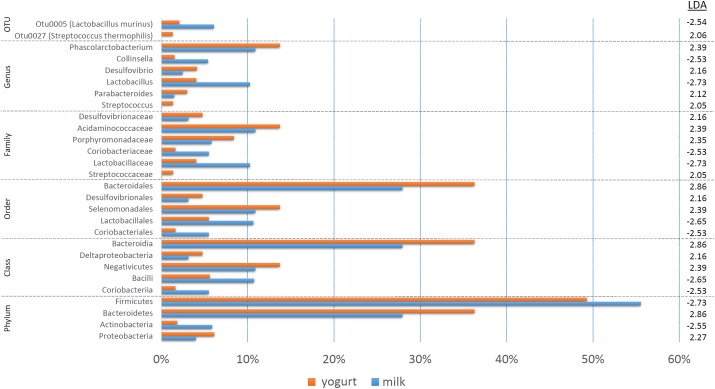
A bar graph of the taxonomic groups indicated by LEfSe analysis to be the most likely to explain the differences between the product types (milk vs. yogurt) (*p* < 0.05, LDA score ≥ 2). The bars represent the abundance of the taxa found in each group. The LDA scores are listed on the right.

The treatment-specific analysis identified that fermentation status (milk vs. yogurt) appeared to influence the composition of the microbial communities. With this in mind, we decided to use LEfSe to compare the combined milk (CM + SM) to the combined yogurt (CY + SY) bacterial communities (Figure 4). Fifteen of the sixteen taxa identified in the comparison of the four treatments, also were significant in the milk vs. yogurt comparison. An additional 12 taxa were also identified as significantly different. Despite attempts to deplete the starter culture bacteria from the yogurts, some viable *Streptococcus thermophilus* (CY: 4.5 × 10^6^ CFU/mL, SY: 1.5 × 10^4^ CFU/mL) were introduced into the rats. These counts are multiple logs lower than the commonly observed abundances in commercial yogurts (10^8^–10^9^ CFU/mL) (Ibrahim and Carr, 2006), however, the viable number of *S. thermophilus* introduced into the rats may have been greater than the initial plate counts as the yogurts sat at room temperature for several hours during the feeding. Thus unsurprisingly, *S. thermophilus* (OTU27) was found in higher abundances in the yogurt, while it was nearly undetectable in the milk samples. Interestingly, *Lactobacillus*, including *Lactobacillus murinus*, was found to be higher in the milks possibly due a greater substrate availability which may have been depleted during fermentation. While *Collinsella* was still found in a higher overall abundance in milk, *C. aerofaciens* (OTU6) was not significantly different in the fermentation status comparison. The genera *Phascolarctobacterium*, *Desulfovibrio*, and *Parabacteroides* were more abundant in yogurt. At a higher phylum taxonomic level, we noted a decrease in the Firmicutes:Bacteroidetes ratio in the yogurt along with an increase in Proteobacteria and decrease in Actinobacteria.

### Product Species Origin Has Little Effect on Cecal Microbial Communities

**FIGURE 5:**
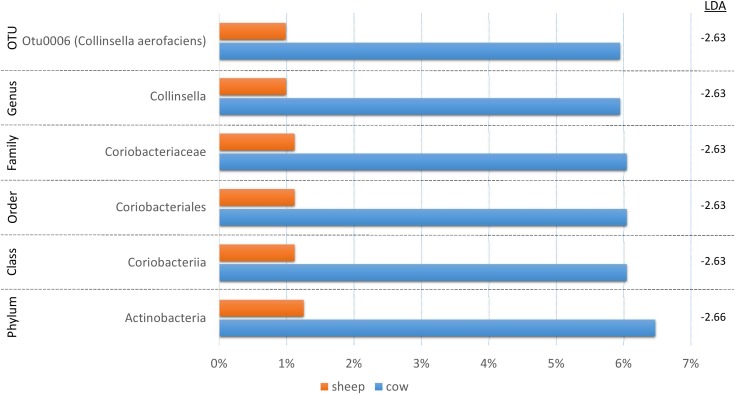
A bar graph of the taxonomic groups indicated by LEfSe analysis to be the most likely to explain the differences in product origins (cow vs. sheep) (*p* < 0.05, LDA score ≥ 2). The bars represent the abundance of the taxa found in each group. The LDA scores are listed on the right.

Since fermentation status appeared to influence the cecal bacterial community composition, we also decided to test if species origin (cow vs. sheep) might affect the bacterial communities using LEfSe (Figure 5). While six taxa were identified as significantly different, the majority of the shift could be linked to a single OTU (*C. aerofaciens*, OTU6) suggesting a lesser influence of species origin on the overall bacterial community. This taxon was also identified in the treatment analyses and the genus *Collinsella* in the fermentation status analysis, suggesting multiple factors affect its abundance.

### Transit Times Do Not Strongly Correlate With Bacterial Taxa Abundance

A parallel study run on the same rats, measured gut transit to determine if the different dairy products affected transit times. It was reported that transit was altered due to treatment method. Those effects appeared to be more related to the origin of the product (cow vs. sheep), than whether they were fermented or not (Dalziel et al., 2018). To determine whether the observed changes in transit scores could be linked to the bacterial communities, we performed regression analysis on the top 100 OTUs, utilizing the combined samples for greater statistical power. Only a single weak significant negative correlation was discovered (*r* = -0.3722, *p* = 0.015) between *Ruminococcaceae* UCG-014 (OTU17) (mean abundance = 1.5%) and total transit score, suggesting abundance of specific taxa was not strongly associated with gut transit (Supplementary Figure S3).

## Discussion

Here we report novel findings of the impact of unfermented and fermented milk (yogurt) from cows and sheep on the rat cecal bacterial composition. The use of healthy rodent models in our study allowed us to control both dietary and environmental factors that would be difficult to implement in human studies and removes confounding factors that make data interpretation more difficult. We showed that the effects of fermentation appeared to have a moderate influence on the composition on the gut microbiota, while animal species origin of the drinks had a small impact. This is in contrast to the parallel gut transit study in which dairy species origin was the dominant influence (Dalziel et al., 2018).

While consumption of fermented milk products containing active bacterial cultures has frequently shown increased recovery of those bacteria within the gut microbiota (Mater et al., 2005; Elli et al., 2006; Oozeer et al., 2006), any effects on the rest of the gut microbiota are less well characterized. Few studies have looked at the effects of fermented milks on the gut microbiota in healthy individuals, and particularly absent are comparisons between unfermented and fermented products. We observed differences in bacterial community composition between consumption of milk and yogurt that could be linked to both the fermentation starter culture (*S. thermophilis*) and other gut community members. Although the viable bacteria in the yogurt starter culture were heavily depleted or eliminated, non-viable/dead cells still may have influenced the composition of the gut microbiota. In contrast, a study in healthy human twins consuming fermented cow milk showed no detectable effect on bacterial community composition (McNulty et al., 2011), although the number of reads per sample was small (1,640 reads), suggesting a lack of depth to detect differences. Other investigations in healthy humans have reported similar results for yogurt (Filteau et al., 2013).

Studies that have compared the effects of both fermented and unfermented milks have been done in both humans and rodents, but they have used patients or rodent models that have or mimic gut dysfunction such as irritable bowel syndrome (IBS) or IBD (Veiga et al., 2010, 2014; Rooks et al., 2014). Rodent studies comparing fermented and unfermented cow milk treatments identified increases in *Desulfovibrio* and Proteobacteria in fermented milk-treated mice and increases in *Lactobacillus* in the unfermented milk-treated mice that match with results observed in our study (Veiga et al., 2010; Rooks et al., 2014). While increases in Proteobacteria and *Desulfovibrio* have been observed in several disease states vs. healthy subjects, we cannot yet discern if and what taxonomic load might represent or lead to a disease status (Gibson et al., 1991; Rowan et al., 2010; Shin et al., 2015). Most *Lactobacillus* are generally thought to be commensal or indicative of a healthy gut microbiota (Fijan, 2014). We did not observe an increase in *Anaerostipes* and *Eubacterium* or decrease in *Enterobacteriaceae* in the fermented milk as compared to unfermented milk. A human study identified an increase in *Roseburia inulinivorans* in a fermented milk treatment compared to the unfermented milk treatment gut bacterial communities (Veiga et al., 2014). Butyrate producers, such as *R. inulinivorans*, may play an important role in preventing GI disorders such as Crohn’s disease (Takahashi et al., 2016).

In our study, we also observed an increase in *Phascolarctobacterium* and *Parabacteroides* in the yogurts. At a higher phylum taxonomic level, we saw a decrease in the Firmicutes:Bacteroidetes ratio in the yogurts along with a decrease in Actinobacteria. *Phascolarctobacterium* has been correlated with positive mood so may play a role in the gut-brain axis (Li et al., 2016). *Phascolarctobacterium* is also a known producer of short-chain fatty acids, including butyrate (Lecomte et al., 2015), so it may mirror some of metabolic effects induced by the increase in *R. inulinivorans* observed in the previously mentioned study (Veiga et al., 2014). A decrease in the Firmicutes:Bacteriodetes ratio has previously been associated with a decrease in body mass index (BMI) (Ley et al., 2006; Turnbaugh et al., 2009). It is unsurprising to note differences in the results between our study and others, as differences in methodology (e.g., diseased vs. healthy host, mice vs. rats vs. human host, fecal vs. cecal samples, 16S vs. shotgun sequencing) substantially influence the outcome. Another factor that may have influenced the results, is that powdered milks were utilized in this study while raw or pasteurized fresh milk have been studied previously.

The fecal microbiota has previously been shown to differ between rats fed cow and other mammals’ milks (e.g., yaks, camels) (Wen et al., 2017). Our analysis also indicated differences in taxonomic abundances between cow and sheep milk, but overall the effects of milk origin were less pronounced than fermentation. The cow milk treatment group was distinct, as it exhibited treatment-specific effects not seen in the other groups. It also showed the greatest intragroup variability which indicates that cow milk may cause more variable gut microbial communities. This suggests particular substrates present in cow milk may trigger a non-uniform response on bacterial communities. Since these effects were not noted in the cow yogurt treatment, fermentation seemingly removes this result. It also appears to be animal species specific, as it was not seen in either sheep treatment group. To help further illustrate the variability in the cow milk treatment group, a pair-wise Morisita-Horn dissimilarity index matrix was generated in mothur (Supplementary Figure S4). Variability (e.g., SM44), was also observed in other treatment groups but was limited as compared to the cow milk treatment samples (Supplementary Figure S4). The high intragroup variability among cow milk samples, also led us to reconfirm the LEfSe results by removing the extreme outliers and performing a one-way ANOVA (*p* < 0.05) on the bacterial abundances (e.g., C*ollinsella*, *Acidaminococcaceae*). The differences remained significant with or without the outliers.

Bacteria of particular interest from this research included *Collinsella* and *Lactobacillus*. The abundance of *Collinsella* appeared to be influenced by both fermentation (milk vs. yogurt) and species origin (cow vs. sheep) and was found in higher abundance in milk (as compared to yogurt from same animal species), particularly cow milk. *Collinsella aerofaciens*, generally considered a gut commensal, is commonly found in the human intestine (Rajilić-Stojanović and de Vos, 2014). Increased *Collinsella* abundance has been associated with both positive and negative health conditions (Malinen et al., 2010; Joossens et al., 2011; Lambeth et al., 2015; Chen et al., 2016), but there is really no consensus of its health effects. Further study is needed as it is not well characterized and has been reported in very low abundance, including those linked to health outcomes. *C. aerofaciens* is known to be able to ferment a range of different carbohydrates, including starches (Kageyama et al., 1999), and there is evidence that it plays a major role in gut lactose fermentation (Kovatcheva-Datchary, 2010). Studies have linked the use of fructooligosaccharides (FOS) and galactooligosaccharides (GOS) to increased activity (Tannock et al., 2004) or abundance (Dewulf et al., 2013; Azcarate-Peril et al., 2017). In a 15 member human gut model in gnotobiotic mice, *C. aerofaciens* was reduced after addition of fermented milk bacterial strains, possibly due to competition with lactic acid bacteria (McNulty et al., 2011).

Lactobacilli are capable of metabolizing a wide range of oligosaccharides and some are considered probiotic (Gänzle and Follador, 2012). *Lactobacillus* sp. have been linked to a number of beneficial effects including improvement in depression symptoms and gut function (Verna and Lucak, 2010; Wallace and Milev, 2017). Although lactic acid bacteria such as *Lactobacillus* are often associated with yogurt (Adolfsson et al., 2004), this may be due to their delivery as live fermentation starter cultures rather than actual growth stimulation of lactobacilli *in vivo*. We found increased abundances of *Lactobacillus* in both cow and sheep milk treatments as compared to their yogurt counterparts which may have been influenced by differences in substrate availability. Lactose can decrease more than 50% post-fermentation (yogurt vs. milk) (Alm, 1982) and the milks used in this study also differed in lactose concentrations (cow milk 3.6%, sheep milk 2.2%). The by-products of lactose fermentation (e.g., lactic acid, ethanol) in yogurt by beta-galactosidases, may be linked to our observed increase in *Desulfovibrio* which can use lactate as an electron acceptor (Price et al., 2014). In this study, there appears to be additional factors at play since changes in abundance of *Collinsella*, another lactose utilizer, do not parallel *Lactobacillus*. Lactic acid bacteria in starter cultures also utilize proteolysis to help successfully drive the fermentation process (Savijoki et al., 2006). This has downstream effects on the availability of types of substrates in fermented products, such as peptides, which could influence microbial abundances (Raveschot et al., 2018).

Bacterial strains exhibit individual preferences for nutrients that affect their ability to compete for specific resources (Fischbach and Sonnenburg, 2011). Both milk species origin and fermentation status influence the availability of nutrients and bioactive compounds in milk products (Pessione and Cirrincione, 2016; Balthazar et al., 2017). Raw sheep milk has a greater amount of protein, fat, and total energy compared to cow (Balthazar et al., 2017). The concentration of vitamins, minerals, and lactose concentrations can vary between animal species (Balthazar et al., 2017). In this study, the use of skim milks reduced the influence of absolute fat. While the milks used in this study were also balanced for protein concentration, the types of proteins in cow and sheep milk still differ and may have influenced the composition of the cecal microbiota.

While lactose is the only major carbohydrate found in cow and sheep milk, the concentrations of the different types of protein fractions varies greatly. There is roughly an 80:20 ratio of total casein and whey proteins in both species, but sheep milk contains mostly β-casein (∼62%) while cow milk has 50% α-casein (40% being αS1-casein) (Balthazar et al., 2017). Cow milk only contains half the β-casein of sheep but 6× more αS1-casein. Specific types of casein have been previously shown to stimulate growth or particular functions, such as biofilm formation, in certain lactic acid bacteria (Azuma et al., 1984; Smid et al., 1991; Varhimo et al., 2011). Amino acid composition also varies among protein fractions types (e.g., α/β-casein, whey) and by animal origin (Gordon et al., 1949; Rafiq et al., 2016). The most abundant amino acid fermenters in the small intestine include genera from *Clostridiales*, Proteobacteria, and the *Bacillus*-*Lactobacillus*-*Streptococcus* group (Dai et al., 2011). While we didn’t observe any substantial changes in these groups associated with animal origin, we did observe changes in Proteobacteria, *Lactobacillus*, and *Streptococcus* in the yogurts that may have been influenced by the availability of amino acids. The changes in lactic acid bacterial abundance are more likely due the difference in availability of casein between milk and yogurt. The lactic acid bacteria used in yogurt starter cultures can have casein-specific proteases which may break down casein during the fermentation process (Liu et al., 2010; Atanasova et al., 2014); this would leave less available to be delivered to the gut microbial community.

Bioactive compounds, such as peptides derived from proteolysis during fermentation, represent another mechanism by which bacterial communities can be influenced. Bioactive peptide composition and activity has been shown to differ between ruminants (e.g., cow, sheep, etc.) following *in vitro* digestion of milk (Tagliazucchi et al., 2018). Many bacterial starter and adjunct cultures used in yogurt manufacture are known to induce the release of bioactive peptides and bacterial strain influences which bioactive peptides are produced (Nguyen et al., 2015). In the parallel study, the number of different bioactive peptides present in milk increased with fermentation (Dalziel et al., 2018). Bioactive peptides from both whey and casein protein fractions of cow milk have been previously shown to stimulate the growth of *Bifidobacterium* and *Lactobacillus* (Azuma et al., 1984; Idota et al., 1994). Other milk peptides, such as lactoferrin, are known to have antimicrobial activity while their derivatives are capable of stimulating bifidobacteria growth (Liepke et al., 2002). The increased diversity and availability of these peptides following fermentation in our study, may have influenced the composition of the microbial community as some of the observed taxonomic changes are known to be influenced by bioactive peptides. In the parallel study, species origin affected total gut transit time (sheep faster than cow) while fermentation did not (milk and yogurt from same origin species were not different) (Dalziel et al., 2018). We did not note any correlations between bacterial abundance and transit time that appeared to be influenced specifically by origin species or fermentation status. A peptide analysis run in the parallel study, identified a β-casomorphin-7 peptide in both the cow milk and yogurt which may have contributed to slower transit (Dalziel et al., 2018).

Complex bacterial communities are not driven by simple direct substrate to strain-specific utilization dynamics, but rather complex interactions such as competition and cross-feeding that function more like a metabolic network (Fischbach and Sonnenburg, 2011). These types of microbial interactions may have driven observed changes in bacterial community composition in this study due to both origin species (composition of the milks) effects and fermentation. We currently lack an understanding of the outcomes of competition and cooperation for specific nutrients among gut microbial communities in their native environment, so it is difficult to predict the size and direction of their impact.

## Conclusion

In summary, this study offers insights into the effects that fermented milk (yogurt) has on the gut microbiota as compared with unfermented milk in a healthy host. It also examined how the differences in animal origin of the milk (cow vs. sheep) might influence gut bacterial communities. We hypothesized, based partially on data from the parallel study, that animal origin would drive gut microbial composition to a greater extent than fermentation. However, while animal origin only impacted the abundance of a single genus (*Collinsella*), fermentation appeared to have a greater effect. The cow milk treatment was shown to produce changes in taxa and wider intra-treatment variation not observed in the other treatment groups. Of particular note, the abundance of *Collinsella aerofaciens* was much higher in the CM treatment than other groups. This may reflect a uniqueness in the availability of substrates and microbial interactions, including cross-feeding, that allowed it to flourish. Future studies looking at the effects of fermentation are needed to gain a greater understanding behind the microbial metabolic processes taking place by measuring bacterial metabolites and surveying microbial gene capability through shotgun metagenomics or RNA-Seq.

## Data Availability

The sequencing data has been deposited into the NCBI SRA archive under Bioproject # PRJNA473571.

## Author Contributions

JD designed the study. ER analyzed and interpreted the data and wrote the manuscript. ER, EA, NR, and JD were involved in manuscript discussion and revision and approved the final version.

## Conflict of Interest Statement

The authors declare that the research was conducted in the absence of any commercial or financial relationships that could be construed as a potential conflict of interest.
