# Supplementary material for: The Effects of Unfermented and Fermented Cow and Sheep Milk on the Gut Microbiota
Source: Front Microbiol. 2019 Mar 6;10:458. doi: 10.3389/fmicb.2019.00458 (PMC6423907; doi:10.3389/fmicb.2019.00458)
Supplement: Supplementary file 4 [file Data_Sheet_1.PDF]

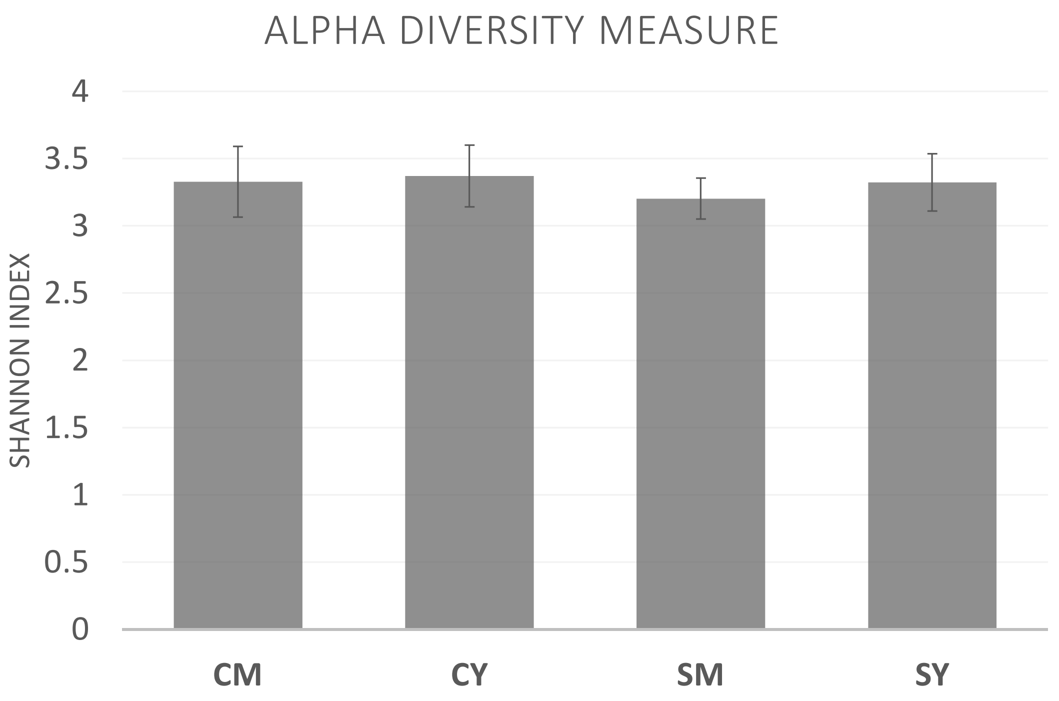

Figure S1. The average Shannon diversity indices for the four dairy drink treatment groups. The error bars indicate the standard deviation.

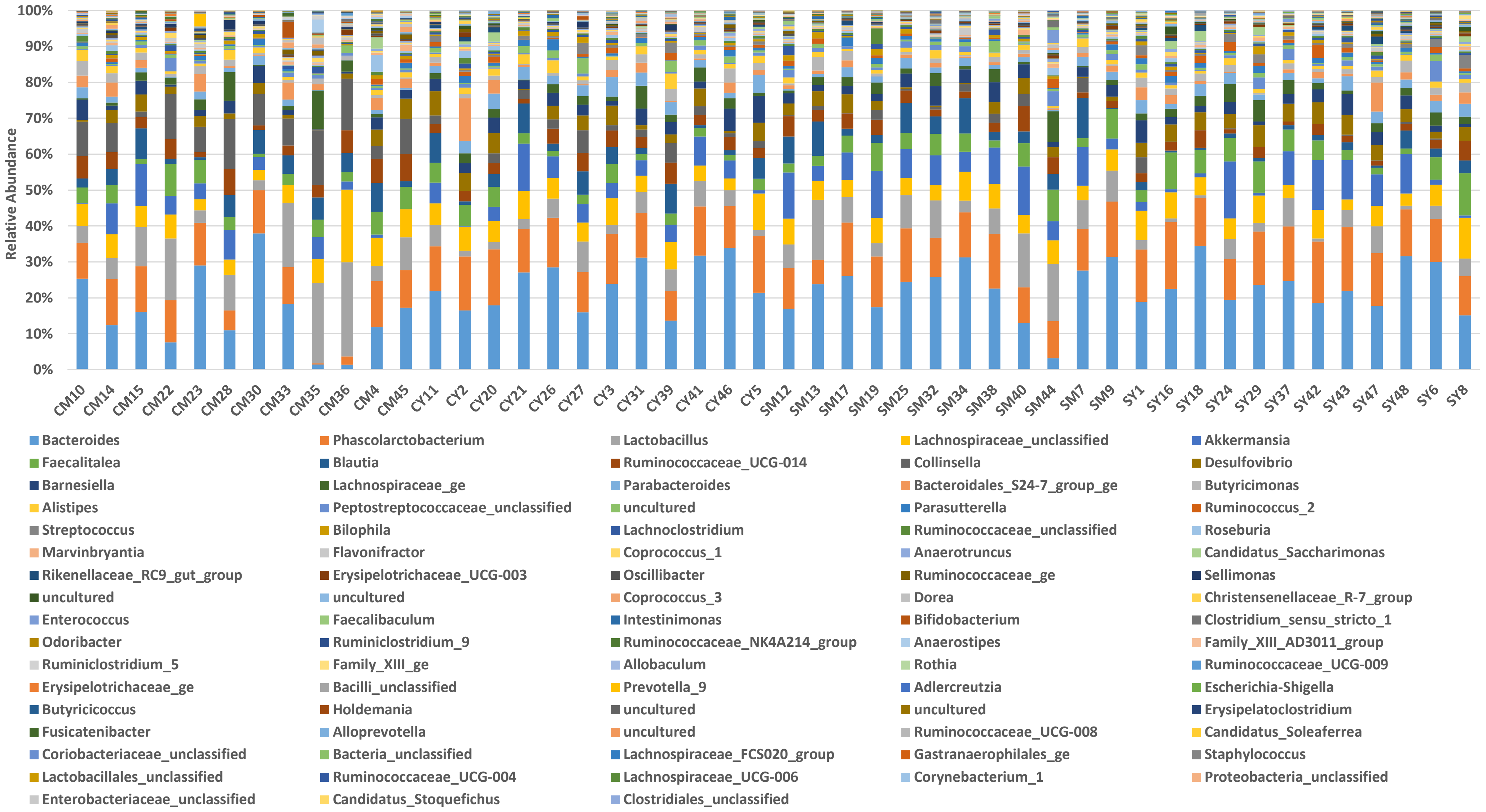

Figure S2. Genus level taxonomic graph representing the relative abundance of bacterial taxa found in each individual rat (cow milk (CM), cow yogurt (CY), sheep milk (SM), sheep yogurt (SY)).

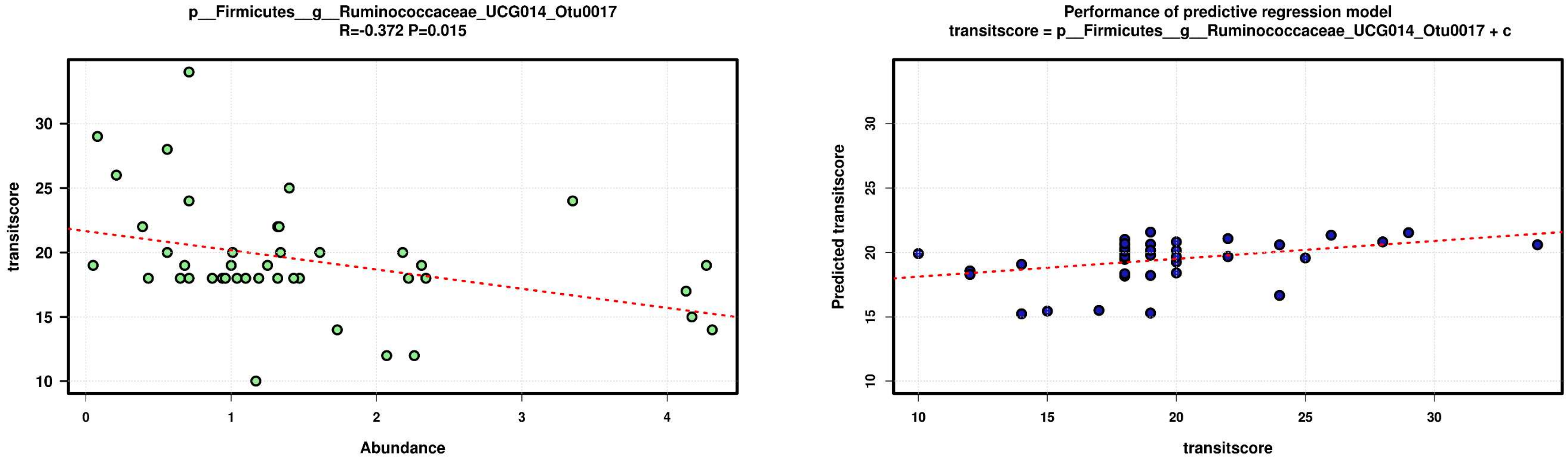

Figure S3. Regression analysis of gastrointestinal transit score and *Ruminococcaceae* UCG-014 (OTU17).

	CM10	CM14	CM15	CM22	CM23	CM28	CM30	CM33	CM35	CM36	CM4	CM45
CM10	0.00	0.24	0.29	0.31	0.09	0.31	0.12	0.16	0.61	0.66	0.32	0.11
CM14	0.24	0.00	0.11	0.17	0.26	0.18	0.34	0.22	0.46	0.54	0.19	0.17
CM15	0.29	0.11	0.00	0.29	0.27	0.26	0.30	0.27	0.55	0.59	0.25	0.29
CM22	0.31	0.17	0.29	0.00	0.36	0.19	0.47	0.21	0.27	0.40	0.27	0.19
CM23	0.09	0.26	0.27	0.36	0.00	0.39	0.06	0.21	0.67	0.74	0.38	0.16
CM28	0.31	0.18	0.26	0.19	0.39	0.00	0.42	0.30	0.17	0.41	0.38	0.29
CM30	0.12	0.34	0.30	0.47	0.06	0.42	0.00	0.26	0.73	0.75	0.44	0.24
CM33	0.16	0.22	0.27	0.21	0.21	0.30	0.26	0.00	0.46	0.54	0.33	0.13
CM35	0.61	0.46	0.55	0.27	0.67	0.17	0.73	0.46	0.00	0.31	0.62	0.51
CM36	0.66	0.54	0.59	0.40	0.74	0.41	0.75	0.54	0.31	0.00	0.62	0.59
CM4	0.32	0.19	0.25	0.27	0.38	0.38	0.44	0.33	0.62	0.62	0.00	0.23
CM45	0.11	0.17	0.29	0.19	0.16	0.29	0.24	0.13	0.51	0.59	0.23	0.00

SM12	SM13	SM17	SM19	SM25	SM32	SM34	SM38	SM40	SM44	SM7	SM9	
0.00	0.15	0.11	0.10	0.08	0.13	0.15	0.09	0.12	0.37	0.10	0.25	SM12
0.15	0.00	0.13	0.27	0.10	0.11	0.12	0.16	0.22	0.42	0.09	0.17	SM13
0.11	0.13	0.00	0.10	0.06	0.06	0.09	0.05	0.18	0.42	0.10	0.09	SM17
0.10	0.27	0.10	0.00	0.11	0.13	0.18	0.09	0.13	0.36	0.15	0.22	SM19
0.08	0.10	0.06	0.11	0.00	0.05	0.05	0.06	0.17	0.38	0.04	0.12	SM25
0.13	0.11	0.06	0.13	0.05	0.00	0.06	0.07	0.21	0.43	0.07	0.08	SM32
0.15	0.12	0.09	0.18	0.05	0.06	0.00	0.10	0.29	0.53	0.04	0.09	SM34
0.09	0.16	0.05	0.09	0.06	0.07	0.10	0.00	0.14	0.38	0.09	0.14	SM38
0.12	0.22	0.18	0.13	0.17	0.21	0.29	0.14	0.00	0.22	0.20	0.35	SM40
0.37	0.42	0.42	0.36	0.38	0.43	0.53	0.38	0.22	0.00	0.50	0.55	SM44
0.10	0.09	0.10	0.15	0.04	0.07	0.04	0.09	0.20	0.50	0.00	0.15	SM7
0.25	0.17	0.09	0.22	0.12	0.08	0.09	0.14	0.35	0.55	0.15	0.00	SM9

	CY11	CY2	CY20	CY21	CY26	CY27	CY3	CY31	CY39	CY41	CY46	CY5
CY11	0.00	0.18	0.07	0.12	0.11	0.08	0.09	0.16	0.13	0.13	0.26	0.10
CY2	0.18	0.00	0.08	0.31	0.20	0.24	0.14	0.23	0.34	0.23	0.29	0.14
CY20	0.07	0.08	0.00	0.21	0.15	0.14	0.11	0.19	0.21	0.18	0.29	0.08
CY21	0.12	0.31	0.21	0.00	0.08	0.17	0.12	0.12	0.22	0.08	0.16	0.17
CY26	0.11	0.20	0.15	0.08	0.00	0.13	0.06	0.07	0.25	0.03	0.08	0.09
CY27	0.08	0.24	0.14	0.17	0.13	0.00	0.16	0.21	0.08	0.17	0.28	0.16
CY3	0.09	0.14	0.11	0.12	0.06	0.16	0.00	0.10	0.26	0.06	0.11	0.08
CY31	0.16	0.23	0.19	0.12	0.07	0.21	0.10	0.00	0.29	0.05	0.09	0.12
CY39	0.13	0.34	0.21	0.22	0.25	0.08	0.26	0.29	0.00	0.30	0.40	0.22
CY41	0.13	0.23	0.18	0.08	0.03	0.17	0.06	0.05	0.30	0.00	0.07	0.13
CY46	0.26	0.29	0.29	0.16	0.08	0.28	0.11	0.09	0.40	0.07	0.00	0.16
CY5	0.10	0.14	0.08	0.17	0.09	0.16	0.08	0.12	0.22	0.13	0.16	0.00

SY1	SY16	SY18	SY24	SY29	SY37	SY42	SY43	SY47	SY48	SY6	SY8	
0.00	0.12	0.20	0.22	0.12	0.12	0.16	0.11	0.12	0.18	0.16	0.21	SY1
0.12	0.00	0.10	0.23	0.04	0.09	0.15	0.12	0.15	0.16	0.11	0.16	SY16
0.20	0.10	0.00	0.27	0.08	0.11	0.20	0.16	0.23	0.09	0.06	0.30	SY18
0.22	0.23	0.27	0.00	0.21	0.09	0.06	0.09	0.11	0.14	0.25	0.30	SY24
0.12	0.04	0.08	0.21	0.00	0.08	0.17	0.14	0.16	0.15	0.07	0.17	SY29
0.12	0.09	0.11	0.09	0.08	0.00	0.07	0.04	0.09	0.06	0.08	0.24	SY37
0.16	0.15	0.20	0.06	0.17	0.07	0.00	0.04	0.09	0.11	0.22	0.30	SY42
0.11	0.12	0.16	0.09	0.14	0.04	0.04	0.00	0.06	0.07	0.15	0.30	SY43
0.12	0.15	0.23	0.11	0.16	0.09	0.09	0.06	0.00	0.13	0.20	0.27	SY47
0.18	0.16	0.09	0.14	0.15	0.06	0.11	0.07	0.13	0.00	0.10	0.36	SY48
0.16	0.11	0.06	0.25	0.07	0.08	0.22	0.15	0.20	0.10	0.00	0.22	SY6
0.21	0.16	0.30	0.30	0.17	0.24	0.30	0.30	0.27	0.36	0.22	0.00	SY8

Figure S4. Moristia-Horn community structure dissimilarity index from pair-wise comparison of intra-group sample bacterial communities. Green indicates more similar community structures while red is more dissimilar.
